# Cardiovascular Computed Tomography Angiographic Assessment of Simple Cardiac Shunts in Adults

**DOI:** 10.31083/RCM43059

**Published:** 2025-11-27

**Authors:** Dhruvil Patel, Douglas Corsi, Anmol Kustagi, Aeos Gaea Baldevia, Abhijay Shah, Lorena Doctor, Aliaa Mousa, Ruchika Bhargav, Andrew Mendoza, Sabahat Bokhari, Kameswari Maganti, Partho P. Sengupta, Yasmin S. Hamirani

**Affiliations:** ^1^Department of Medicine, Division of Cardiovascular Diseases and Hypertension, Rutgers Robert Wood Johnson Medical School, New Brunswick, NJ 08901, USA

**Keywords:** congenital heart disease, cardiovascular computed tomography angiography, patent foramen ovale, atrial septal defects, ventricular septal defects, patent ductus arteriosus, anomalous pulmonary venous return, coronary artery fistulas, unroofed coronary sinus, 3D printing

## Abstract

Congenital heart disease (CHD) is increasingly detected in cardiac imaging. Effective management of CHD requires thorough imaging of the heart and circulation, extending beyond simple anatomical identification. Cardiovascular computed tomography angiography (CCTA) provides rapid imaging, high spatial resolution, and precise visualization of three-dimensional vascular structures, while offering strong multi-planar reconstruction capabilities at sub-millimeter resolution and a wide field of view. These features enable CCTA to overcome the challenges faced by other imaging modalities. Thus, this review highlights the advantages of CCTA in evaluating simple cardiac shunts in adult congenital heart disease pre- and post-intervention.

## 1. Introduction

The diagnosis of adult congenital heart disease (CHD) is on the rise. Time trend analyses indicate that the global prevalence of CHD has been increasing by 10% every 5 years since 1970 [1, 2, 3]. This surge has been accompanied by increased accessibility to and advancements in imaging technologies that have led to prolonged patient survival, shifting mortality from a bimodal age distribution to a distribution skewed toward older age [4]. Cardiovascular computed tomography angiography (CCTA) is essential for diagnosis, procedural guidance, and long-term follow-up of adult patients with CHD.

The benefits of computed tomography (CT) imaging include an inherently high spatial resolution, excellent air-tissue contrast, and multiplanar reconstruction capabilities. CT’s expansive field of view provides high-resolution and precise imaging of the heart, mediastinum, pulmonary structures, and vascular systems, which is instrumental in identifying concomitant pathologies and assessing the pulmonary vasculature in detail [5]. Unlike magnetic resonance imaging (MRI), which provides higher temporal resolution, CT imaging offers higher spatial resolution. It can identify and characterize defects, visualize improper shunting at the microscopic imaging level, assess the three-dimensional (3D) spatial area of transcatheter interventions and sizing, support surgical intervention, create 3D modeling, and help select appropriate candidates for percutaneous device placement [5]. Moreover, this imaging modality has improved with the increasing number of slices, development of dual-energy, and the most modern photon-counting detector (PCD)-CT system [6, 7]. Innovation in CT technology has allowed for improved image quality with lower radiation exposure and a reduction in contrast dosage. These advances in CCTA have established its place as a vital diagnostic tool [6, 7].

In this paper, we aim to examine the value of cardiac CT imaging in diagnosing adult CHD, focusing on the noninvasive interrogation of simple shunts pre- and post-intervention. This review will explore common CHDs such as patent foramen ovale (PFO), atrial septal defects (ASD), ventricular septal defects (VSD), patent ductus arteriosus (PDA), and types of anomalous pulmonary venous return (APVR). In addition, we will also review rare defects such as coronary artery fistulas (CAFs) and unroofed coronary sinus (UCS). Complex adult congenital heart diseases will not be discussed in this review.

## 2. CCTA Protocols to Evaluate Simple Intracardiac Shunts

Table 1 (Ref. [8, 9, 10, 11, 12, 13, 14, 15, 16]) presents a comprehensive analysis of validated CCTA acquisition protocols documented in the literature for the evaluation of simple congenital cardiac shunts and incorporates contemporary scanner technologies and their specific parameters. Protocol optimization for individual shunt lesions is further elaborated in their respective dedicated sections.

**Table 1.** **Cardiac CT angiography protocols used in literature for assessment of simple intracardiac shunts in adults**.

Intracardiac shunt	Scanner type used	Minimum scan window	Slice thickness (mm)	Electrocardiogram (ECG)-gating	Peak kilovoltage (kVp)	Tube current (mA or mAs)	Optimal attenuation (HU)	Optimal contrast volume (mL)	Contrast rate (mL/s)	Delayed imaging (y or n)
Patent foramen ovale (PFO) [9, 10]	64 slice	Carina to diaphragm; coronal oblique projections through interatrial septum	0.9 mm	Retrospective, effective radiation dose around 2–6 mSv	120–140 kVp	600–900 mA		60–120 mL iodine contrast agent and iomeprol followed by 50 mL saline solution	5–6 mL/s injected into an antecubital vein through an 18G–20G catheter	No
	320 slice		0.5 mm	Retrospective, effective radiation dose around 2–6 mSv	100–135 kVp	400–600 mA				
Atrial septal defects (ASD) [11]	64 slice	Carina to diaphragm	0.5 mm	Retrospective preferred	100–120 kVp	Automatically adjusted for even potential; 250–865 mAs	130 HU	55–75 mL contrast followed by 50 mL of mixed 80:20 saline/contrast	5 mL/s through the antecubital vein via an 18G cannula	Yes, scanning initiated 4 seconds after attenuation has reached threshold in region of interest (ROI): descending aorta
								70–80 mL of contrast without saline bolus	3.5 mL/s through the antecubital vein via an 18G cannula	
Patent ductus arteriosus (PDA) [8]	Dual-source	Aortic arch to diaphragm	0.6 mm	Retrospective preferred	100–120 kVp	220–330 mAs	140 HU	1.5 mL/kg body weight is injected followed by saline bolus	3–4 mL/s	Yes, used bolus tracking technique to determine imaging delay in ECG-synchronised CT
Ventral septal defects (VSD) [11]	64 slice	Carina to diaphragm	0.5 mm	Retrospective preferred	100–120 kVp	Automatically adjusted for even potential; 250–865 mAs	130 HU	55–75 mL contrast followed by 50 mL of mixed 80%:20% saline/contrast solution	5 mL/s through the antecubital vein via an 18G cannula	Yes, scanning initiated 4 seconds after attenuation has reached threshold in ROI: descending aorta
								70–80 mL of contrast without a saline bolus	3.5 mL/s through the antecubital vein via an 18G cannula	
Unroofed coronary sinus (UCS) [12, 13]	Single-source 64 slice and 40-row dual-source	Carina to diaphragm	0.625–0.75 mm	Retrospective preferred	100–120 kVp	200–550 mAs	100 HU	1.0–1.2 mL per patient kg, iohexol 350 mgI/mL or iopromide 370 mgI/mL was injected, following with 40 mL sterile saline at same rate	Double-head power injector (DHPI) used to inject contrast media at a flow rate of 4.0–5.0 mL/s through a 20G trocar in an antecubital vein	Yes, 6 s delay as bolus-tracking was used in ROI: ascending aorta
Persistent left superior vena cava (PLSVC) [14]	64 slice	Carina to diaphragm	0.625 mm	Retrospective preferred	120 kVp	600 mAs		50 mL of contrast solution 350 mg/mL followed by 50 mL of saline flush	4 mL/s	Yes
Coronary artery fistulas (CAF) [15]	Dual-source	Carina to diaphragm	0.6 mm	Retrospective and Prospective	100–120 kVp	320 mAs	120 HU	80 mL of iopromide followed by 50 mL injection of 85%:15% saline-contrast solution	5 mL/s	Yes, used bolus tracking in the ascending aorta and scan delay was 9 s
Anomalous pulmonary venous return (APVR) [16]	3rd-generation dual source	Aortic arch to diaphragm	0.6 mm	Prospective	80 kVp	270 mAs		Non-ionic iodinated contrast (1.5–2.0 mL/kg)	Administered via peripheral IV using DHPI at 1.0–4.0 mL/s	No, CT acquisition was manually triggered when optimal contrast opacification within pulmonary vessels was achieved on visual monitoring sequence

CCTA requires careful preparation and individualized assessments. Healthcare providers must conduct a thorough evaluation of the patient’s medical history, including allergies to contrast agents and existing renal conditions, to mitigate potential risks associated with contrast use. Adults generally possess larger and more accessible veins, facilitating the placement of larger intravenous (IV) lines, often 18-gauge, which allows for more efficient contrast administration compared to pediatric patients. The volume of iodine-based contrast used is calculated based on the patient’s weight and renal function, ensuring a tailored approach to avoid complications like nephrotoxicity.

When performing a CCTA, considerations should be made for radiation dose, body mass index (BMI), and heart rate. Adherence to the “as low as reasonably achievable” (ALARA) principle is crucial to minimize radiation exposure. This involves employing optimal imaging parameters, including kilovoltage (kV) and milliampere (mA) settings. For some conditions additional z coverage is required. Patients with a higher BMI possess more adipose tissue, which necessitates increased kV and tube current to maintain diagnostic image quality. Expert consensus from the American College of Cardiology (ACC)/Heart Rhythm Society (HRS)/North American Society of Cardiovascular Imaging/Society for Cardiovascular Angiography and Interventions (NASCI)/Society for Cardiovascular Angiography and Interventions (SCAI)/Society of Cardiovascular Computed Tomography (SCCT) recommends BMI-adjusted kV settings during scans: 80 kV for a BMI <21 kg/m^2^; 100 kV for a BMI between 21–29 kg/m^2^; and 120 kV for a BMI ≥30 kg/m^2^. For patients with a BMI ≥40 kg/m^2^, a maximum tube potential of 140 kV may be required to obtain diagnostic-quality images [17]. Additionally, most modern scanners are equipped with tube current modulation, which adjusts the tube current based on the estimated body thickness derived from the topogram, resulting in a radiation reduction of up to 20% [17]. Advanced dose reduction strategies, such as axial-sequential acquisition, high-pitch helical techniques, and prospective gating, are utilized to enhance image quality while maintaining minimal radiation exposure. Retrospective gated imaging can result in a radiation dose ranging from 2 to 6 mSv [17]. This dose may be higher in patients with elevated BMI, even when dose modulation techniques are applied. In contrast, prospective gating significantly reduces radiation exposure by 50%, lowering the dose to between 1 and 3 mSv in adults [8]. Recent advancements in PCD-CT show potential for improved dose efficiency, ultra-high resolution (up to 0.2 mm), and enhanced contrast-to-noise ratio. PCD-CT can achieve radiation dose reductions of 30–66% compared to traditional energy-integrating detector CTs (EID-CT) [18]. This is particularly beneficial for overweight and obese individuals, who experience a dose reduction of 27% to 34% while maintaining favorable signal-to-noise and contrast-to-noise ratios [19]. Although PCD-CT is still in its early stages, these findings suggest significant promise for the field of CHD.

Heart rate is another crucial factor in CCTA procedures. Prospective gated imaging is most effective when the heart rate is below 65–70 beats per minute, as higher heart rates can result in diminished image quality. To manage this, beta-blockers may be administered to achieve the desired heart rate. However, if the heart rate remains excessively high or irregular, retrospective gating or padding may be necessary, which can increase radiation exposure for the patient [17, 20]. In some situations, retrospective gating is essential for gathering data throughout the cardiac cycle to assess chamber volumes and perform pulmonary-to-systemic blood flow ratio (Qp/Qs) calculations. In such cases, reducing the tube voltage from 120 kV to 100 kV and utilizing automatic tube current modulation can significantly decrease radiation doses, provided the image quality allows.

Summary of essential CCTA measurements and associated defects can be found in Table 2 and individual shunts are discussed in detail below.

**Table 2.** **CCTA findings and measurements for simple intracardiac shunts in adults**.

Congenital heart defect	CT measurements	Associated defects
Atrial septal defect (ASD)	-Defect 3D size and length	-Mitral valve cleft
	-Measurement of size of 4 rims (aortic, posterior, superior and inferior)	-Down syndrome
	-Thickness of the membranous septum	-Interventricular defect
	-Advanced software can be used to assess for interatrial septal puncture site and decide on additional curves needed on the delivery sheath for transcatheter	-Anomalous pulmonary venous return
Ventricular septal defect (VSD)	-Defect number and location	-Double-chambered right ventricle
	-Defect 3D shape, size and length	-Subaortic ridge
	-Cardiac chamber 2D sizing (3 RV and LV volumes in systole and diastole if retrospective data available)	-Gerbode defect
	-Assessing shunt volume/fraction [Qp/Qs]	-Aortic coarctation
	-Relationship/distance of VSD from valves and other heart structures	-Pulmonary hypertension (Eisenmenger syndrome)
	-Presence of IVS aneurysm	
	-Size of pulmonary arteries	
	-Assess for RVH	
Patent ductus arteriosus (PDA)	-Ductal length	-ASD
	-Minimal and maximal diameters of ostia	-VSD
	-Quantification of calcification burden	-Tetralogy of Fallot
	-Assess for RVH	-Pulmonary hypertension (Eisenmenger syndrome)
	-Size of pulmonary arteries and aorta	
Anomalous pulmonary venous return (APVR)	-Diameters of pulmonary vein ostia	-ASD
	-Dimensions of pulmonary venous confluence	-PFO
	-Distance between pulmonary venous confluence and left atrium	-Lung hypoplasia
	-Vertical vein diameters (min and max)	-Dextrocardia
Coronary artery fistulas (CAF)	-Origin of proximal vessel and course of blood flow	-PDA
	-Size and anatomy of distal vessel entry site	-Pulmonary AV fistula
	-Size of receiving cardiac chamber or vessel at distal CAF vessel site	-Ruptured sinus of Valsalva aneurysm
	-RV and PA size	-Prolapse of right aortic cusp with supracristal VSD
Unroofed coronary sinus (UCS) and persistent left superior vena cava (PLSVC)	-Size of CS roof defect	-ASD
	-Ostium of the defect	-VSD
	-CS Index: CS size normalized to body surface area	-Uni-atrial heart
	-Assessment for UCS’ different types based on extent and location of the defect and presence/absence of PLSVC	-Abnormal pulmonary venous drainage
		-Tetralogy of Fallot
		-PLSVC

Abbreviations: CT, computed tomography; 3D, three-dimensional; 2D, two-dimensional; RV, right ventricle; LV, left ventricle; IVS, interventricular septum; Qp/Qs, pulmonary-to-systemic flow ratio; RVH, right ventricular hypertrophy; PFO, patent foramen ovale; PA, pulmonary artery; CS, coronary sinus; AV, arteriovenous; CCTA, cardiovascular computed tomography angiography.

## 3. Patent Foramen Ovale and Atrial Septal Defects 

### 3.1 Patent Foramen Ovale

**Fig. 1.**
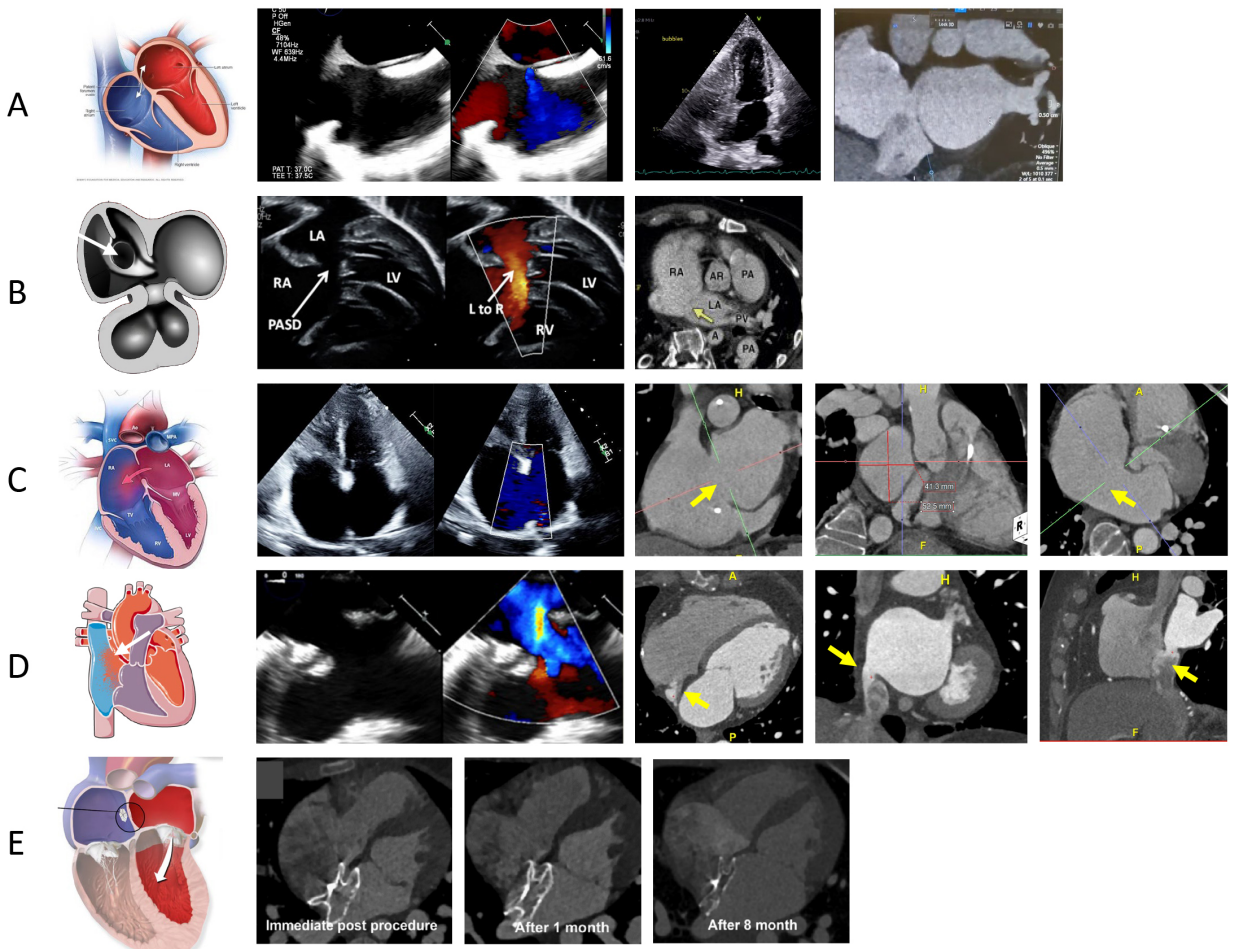
**PFO and ASD evaluation on CCTA [26]**. (A) The left-most image reveals a schematic highlighting the PFO, the middle images are a PFO evaluation on TEE on 2D and Color Doppler, and a TTE with bubble study revealing bubbles crossing from right to left atrium and the right-most image is a CCTA image revealing the presence of PFO tunnel with contrast shunting from left to right. (B) The left image reveals a schematic highlighting primum ASD [27], the middle image is a 2D and Doppler echo assessment of primum ASD [28], and the right image is a CCTA revealing presence of ostium primum defect (arrows) [29]. (C) The left-most image reveals a schematic highlighting the secundum ASD, the middle image is a TTE assessment of secundum ASD, and the last three images on the right are CCTA images evaluating a large secundum ASD with measurement (arrows). (D) The left-most image reveals a schematic highlighting the superior sinus venosus ASD the middle images are TEE assessment of sinus venosus defect, and the last three images on the right are CCTA images revealing presence of an inferior sinus venosus ASD (arrows). (E) The left-most images (schematic of ASD device closure), right three images show morphologic changes of device neo-reendothelialization on CCTA immediately post procedure, after 1 month and after 8 months [30]. LA, left atrium; TEE, transesophageal echocardiography; TTE, transthoracic echocardiography; PASD, primum atrial septal defect.

Patent foramen ovale is a common congenital cardiac anomaly with a prevalence of 27% [21] characterized by a flap-like opening between the atria that persists after birth [5]. While PFOs are often asymptomatic, they become clinically significant in cases such as cryptogenic stroke, migraine with aura, platypnea-orthodeoxia syndrome, transient ischemic attacks, or decompression sickness due to paradoxical embolism [5, 22, 23]. Diagnosis of PFOs traditionally relies on transthoracic or transesophageal echocardiography (TTE or TEE), often performed with an agitated saline solution as a contrast agent [24]. Bubble studies are then used to confirm right-to-left shunting during the Valsalva maneuver [24]. While TEE is considered the standard technique for diagnosing right-to-left shunts that confirm PFOs, the sedation needed to perform the Valsalva maneuver can make it more difficult for some patients [21]. CCTA can provide further characterization of the PFO size, length and confirm shunting, although it is less sensitive since maneuvers to enhance shunting are not performed during the exam [25] (Fig. 1, Ref. [26, 27, 28, 29, 30]).

CCTA findings that are indicative of a PFO include the presence of a channel-like appearance of the interatrial septum (IAS), left-to-right flow of contrast towards the inferior vena cava through the channel [10, 31]. Right-to-left interatrial shunting can be visualized as negative contrast from the septum towards the left atrium [32]. Electrocardiogram (ECG)-gated CT enables retrospective imaging across the cardiac cycle, allows functional evaluation, and identifies anatomical relationships critical for procedural planning [10, 31]. Thus, CCTA can be used for pre-procedural planning for percutaneous PFO closure. Post procedure CCTA allows ruling out any complications secondary to PFO closure including assessing device positioning and impact on coronary arteries [31]. However residual shunts are challenging to evaluate with CCTA due to artifacts related to the closure device [31].

### 3.2 Atrial Septal Defects

Atrial Septal Defects comprise about 10% of CHD [1, 11]. The four main types of ASDs include ostium secundum, ostium primum, sinus venosus, and coronary sinus defects [5] (Fig. 1).

Ostium secundum ASD accounts for 70% of all ASD [5]. Ostium Secundum occurs in the mid atrial septum and corresponds to a defect in the septum primum at the fossa ovalis [1, 5]. Ostium Primum ASD is characterized by a defect in the anterior and inferior part of the IAS where the septum primum fails to fuse with the endocardial cushion at the antero-basal part of the atrial septum [5]. The area of deficiency may result in either anatomically anomalous pulmonary veins (discussed in Section 6), or in some cases, the venous connections are anatomically appropriate but have inappropriate effective drainage. This type of ASD occurs in about 15% of patients with Down Syndrome and is often associated with interventricular defects [5]. Sinus Venosus ASDs (SV-ASDs) also referred to as “sinus venosus defects” account for 10% of all ASDs and represent a deficiency between the interatrial septum and the wall of the superior or inferior vena cava, and in some cases, the pulmonary veins. SV-ASDs, when involving only the systemic and pulmonary veins and not the atrial septum, can be repaired via transcatheter intervention in the modern era [33]. Coronary sinus defects will be discussed separately (see Section 7.2).

CCTA imaging plays a crucial role in the diagnosis and procedural planning for ASD. It allows for detailed evaluation of ASD in multiple orthogonal planes with measurement of the size and shape of the defect, assessment of the surrounding rims, locating associated shunts, and identification of adjacent cardiac structures such as the pulmonary veins, aortic root, and coronary arteries [31, 34]. CCTA offers an adjunct assessment to echocardiography by providing an en face view of the defect, as well as coronal oblique images obtained from 4-chamber and short-axis reconstructions of the atrial septum [34]. CCTA has been found to have sensitivity of 90–96% and specificity of 88–97% for the detection of ASD and a mean discrepancy of 1.1 mm compared to intraoperative sizing, underscoring its reliability and value in both pre- and post-procedural settings [34]. To optimize image quality, a heart rate below 60 beats per minute and a triphasic bolus of contrast are recommended to ensure homogeneous chamber opacification with minimal admixture artifacts [34].

For pre-procedural planning, CCTA can show certain anatomic features that significantly influence the feasibility of device closure. Deficient septal rims have been associated with device erosion, embolization, or instability and may prompt surgical referral [35]. In adult patients, a septal rim of ≥5 mm in all directions is generally considered adequate for transcatheter device closure [35]. Patients with deficient posteroinferior rims carry a higher risk for device embolization towards the inferior vena cava [33]. For this type of rim deficiency, specific measurements such as a defect size/total septum length ratio <0.35, aortic rim/defect size ratio of >0.75, and a posterior inferior rim/defect size ratio >1.0 can be used to predict the success of percutaneous closure [35]. Finally, very large secundum defects (>25 mm in adults), especially those approaching or exceeding the size limits of available occluders, may also present challenges for device-based closure [36]. In these cases, CCTA plays a critical role in accurately assessing defect dimensions and surrounding anatomy to guide appropriate treatment planning [36].

Post-procedurally, CCTA is used to confirm the success of interventions, evaluate the placement of surgical patches or transcatheter devices, and detect complications, such as device embolization, thrombus formation, or residual shunting [37]. No differences have been noted between CCTA and TEE in evaluating success rates of device closure, complications, or ratio of device size to the maximum diameter of the defect. A 2020 study by Zhang *et al*. [33], looked at safety and visibility of transcatheter closure of ASD with just CCTA sizing in 134 patients. In a 2022 study, Kim *et al*. [30] looked at CCTA for assessment of device neo-endothelialization after transcatheter closure as well as for thrombosis or vegetation attached to the device for both bulky and flattened devices. Contrast opacification within the device was identified as complete, partial, and non-opacified. If there was no contrast opacification within the device and the shape of the device was flattened, neoendothelialization was considered complete (Fig. 1). Device thrombosis was defined as the presence of focal low attenuation thickening on the atrial surface of the device. Continued use of CCTA in assessing ASD has established itself to be a comparable and complementary imaging modality to echocardiography.

## 4. Ventricular Septal Defects 

Ventricular septal defects account for approximately 20–30% of all congenital heart conditions [1, 5]. If not addressed promptly and allowed to persist, they may lead to pulmonary arterial hypertension, Eisenmenger syndrome, as well as a compounded risk of developing arrhythmias. VSD closure criteria includes findings of shunt fraction with Qp/Qs of >1.5, pulmonary artery systolic pressure more than 50% of the systemic arteriolar pressure and pulmonary vascular resistance greater than one-third of the systemic resistance [38].

TTE is the first line imaging modality for evaluation of VSDs. However, there are several limitations: (1) it can be difficult to visualize certain types of defects in patients with large body habitus, (2) complex defects can be difficult to visualize, (3) adequate interrogation requires off-axis imaging and (4) is dependent on the expertise of the sonographer [39]. CCTA allows for precise measurements of the defect size, and its impact on cardiac chambers, such as right ventricular enlargement or increased pulmonary blood flow. A slice thickness of ≤0.6 mm is optimal for delineating borders, particularly where surgical patch placement is being considered. For optimal visualization, high-pitch spiral or prospectively ECG-triggered sequential CT angiography is recommended, with contrast opacification during mid-to-late systole. If quantification of blood flow through the defect indicated, retrospectively gated CCTA allows assessment of end-diastolic and end-systolic volumes in the ventricles and Qp/Qs calculation. CCTA can help guide treatment which includes conservative monitoring, transcatheter closure or surgical intervention.

The data from CCTA measurement of acquired VSD dimensions secondary to septal rupture can be extrapolated to congenital VSD evaluation pre-closure. The study from Chen *et al*. [40] in 44 patients depicted that using CCTA to measure shunt activity before the procedure led to much smaller residual shunts after transcatheter closure compared to using echocardiography (median 2.1 mm vs 4.2 mm, *p* = 0.005). The measurements from CCTA also closely matched the size of the occluder that was implanted (r 0.799), but echocardiography measurements do not correlate to the same extent. This can help in choosing the right device, improving the procedure course, and assisting with check-ups after the procedure, especially when looking at residual shunts after VSD device closure [40]. CCTA effectively enables detailed assessment of VSD morphology, offering precise measurements of septal rims tying in relation to adjacent anatomical landmarks. According to He *et al*. [41], CCTA can quantify the subaortic rim very optimally (reported at 3.0 ± 1.5 mm in one series), helping assess the feasibility of intervention by quantifying tissue reserve to facilitate device anchoring. It is generally advised to choose a device size 1–2 mm larger than the measured defect diameter to facilitate stability and minimize residual shunting. Anatomical characteristics significantly influence device selection strategies. Defects with adequate septal rim support (>3 mm) and small dimensions (<5 mm) favor selection of devices sized 1 mm larger than the smallest measured diameter [42]. Conversely, defects characterized by compromised rim support (<4.5 mm) may necessitate alternative approaches, including utilization of Amplatzer muscular occluders sized according to ventricular entry measurements rather than defect diameter [42].

### 4.1 CCTA Characterization of Different Types of VSDs 

**Fig. 2.**
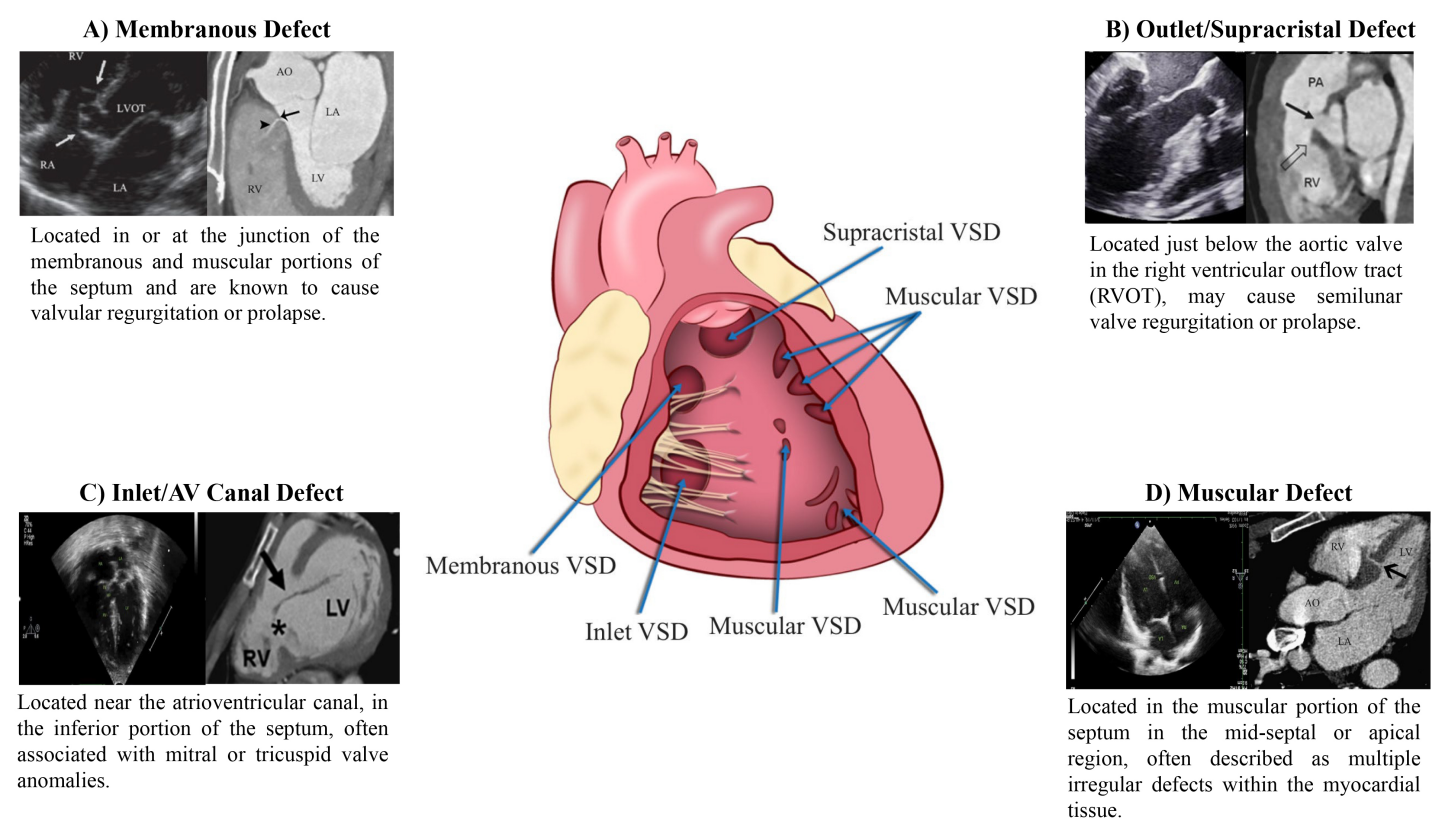
**VSD evaluation on CCTA**. (A) TTE and CCTA images of membranous defect [43]. (B) TTE and CCTA Images of outlet/supra-cristal defects [44, 45]. (C) TTE and CCTA images of inlet/AV canal defect [46]. (D) TTE and CCTA images of muscular defect [5, 46].

The different types of VSDs include membranous defects, muscular defects, outlet or supracristal defects and inlet or atrioventricular canal defects (Fig. 2, Ref. [5, 43, 44, 45, 46]). They can occur in isolation or can also be present in coexistence with other congenital heart defects like ASDs and PDAs [39, 47].

Membranous VSDs are defined by fibrous continuity between the atrioventricular valves at the septum’s posterior-inferior margin [8]. They may extend into the muscular or inlet portions and are often associated with conditions such as double-chambered right ventricle, subaortic ridge, Gerbode defect, and aortic coarctation. Multiplanar and volume-rendered reconstructions assist in pre-surgical planning by detailing the proximity to the aortic root, coronary ostia, and conduction system structures. Muscular VSDs are found in the lower part of the septum. They are small and when multiple small defects are present, they are described as a “Swiss cheesecake” septum appearance. However, 75% heal on their own due to hypertrophy of the surrounding tissue [48]. In selected cases, single distal muscular defects are amenable to device closure due to favorable anatomy. CT imaging should be performed using retrospective ECG-gating with dose modulation to capture full cardiac phases, as muscular VSDs may be more visible in end-systole. Curved planar reconstructions are particularly useful for tracking serpiginous or tunnel-like extensions that may alter interventional strategy. Evaluation of adjacent RV trabeculations is essential to exclude accessory defects or restrictive morphology.

Outlet (supracristal) VSDs, located in the conal septum adjacent to the right ventricular outflow tract, often lead to progressive aortic cusp prolapse with resultant aortic regurgitation. Though uncommon, they pose disproportionate technical difficulty. For accurate characterization, CT imaging should be acquired in mid-systole using ECG-gated arterial-phase acquisition, with bolus tracking placed in the ascending aorta. This allows precise assessment of the spatial relationship between the VSD and the right coronary cusp, as well as quantification of aortic valve distortion. Oblique sagittal reconstructions parallel to the right ventricular outflow tract help evaluate coexisting conal anomalies [49]. Inlet (atrioventricular canal) VSDs also known as endocardial cushion defects, occur near the crux of the heart and are often associated with complete AV septal defects or discordant AV connections. They do not close spontaneously and are not suited for device-based intervention. CT protocol should prioritize retrospective ECG-gating with multiphasic acquisition to assess dynamic atrioventricular valve interaction with the defect. Thin-slice isotropic imaging combined with virtual dissection planes through the AV junction helps define leaflet alignment and AV morphology [8, 50].

While VSDs represent true septal discontinuities, other outpouchings such as ventricular diverticula and aneurysms may mimic them on echocardiography or MRI. Cine CCTA is particularly valuable in distinguishing these entities. A diverticulum typically contracts synchronously with the myocardium and has a narrow neck, while an aneurysm exhibits paradoxical motion and a wide neck. Furthermore, dual-energy CT with iodine mapping allows differentiation of fibrotic, non-enhancing aneurysmal walls from contrast-filled shunt channels [51].

### 4.2 Intra-Procedural Use of CCTA for VSD Closure

Although CT is predominantly employed in the preprocedural phase, CT-guided navigation can assist in real-time device deployment. By providing continuous, high-quality imaging during the procedure, it helps ensure accurate placement of the device; this, in turn, reduces the occurrence of complications such as device migration, device embolization, or incomplete closure [52].

### 4.3 Use of CCTA Post Transcatheter or Surgical Closure of VSD

Previous studies have highlighted that approximately 35% of patients experience residual shunting after VSD repair, with a shunt size of 1.25 mm serving as a reliable predictor of postoperative outcomes [53]. Given its high temporal resolution, CCTA is ideally suited to precisely assess residual shunting, allowing for a detailed evaluation of blood flow across the repaired defect. Patch closure of VSDs can lead to complications such as infection, mechanical failure, or valve dysfunction, particularly in cases where the defect is near the aortic valve. CCTA can assess the integrity of the patch and detect potential displacement, dehiscence, or infection. In addition to assessing the surgical site, CT can also evaluate changes in ventricular size post-repair [54]. A common source of diagnostic confusion in the post-closure setting is the presence of a membranous septal aneurysm with persistent contrast jetting. CCTA plays a pivotal role in differentiating this benign finding from a residual shunt. By analyzing contrast wash-out over multiple phases and evaluating neck morphology, CT can determine whether a patent communication exists or if the contrast pooling is confined to a closed pouch. This distinction is crucial in avoiding unnecessary reintervention and in counseling patients regarding long-term prognosis [55]. However, multimodality imaging is required in some situations due to its specific strengths and weaknesses. Echocardiography detects residual VSD shunts with high accuracy, identifying up to 93% immediately post-closure in a successive manner, with Doppler defining jet direction, velocity, and severity. Cardiac MRI too achieves a sensitivity of 90% mark with phase-contrast and 4D-flow, Qp/Qs quantification with 100% sensitivity and a 93% specificity for clinically significant shunts. CCTA despite providing sub-0.5 mm spatial resolution for anatomical assessment, does not allow adequate evaluation of residual flow across the closure device [56].

## 5. Patent Ductus Arteriosus

Patent ductus arteriosus represents 5% to 10% of all congenital heart disease and is associated with an estimated mortality rate of 1.8% per year in untreated adults [57]. This persistent embryological connection between the left main pulmonary artery and descending thoracic aorta requires prompt identification, particularly for moderate-to-large PDAs, to prevent progression to Eisenmenger syndrome and facilitate appropriate surgical or catheter-based interventions. CCTA has emerged as a vital diagnostic tool for PDA detection and evaluation, with PDA identified as an enhancing structure connecting the left main pulmonary artery to the descending thoracic aorta [58]. Beyond routine detection, CCTA demonstrates value in diagnosing clinically silent PDAs, which often present as incidental findings during routine chest imaging, and in clinical scenarios where echocardiographic evaluation is limited by low shunt flow or severe pulmonary hypertension [58]. One study demonstrated CCTA’s diagnostic superiority over echocardiography for PDA detection, achieving 100% sensitivity and specificity compared to echocardiography’s 93.3% detection rate when validated against cardiac catheterization and surgical findings [59].

For optimal management of PDA, accurate anatomical characterization through advanced imaging is essential for diagnosis as well as guiding therapeutic interventions via the assessment of shunt physiology [60]. The Krichenko classification system categorizes PDA morphology into 5 distinct types based on angiographic appearance [61] (Table 3, Ref. [61, 62, 63]). Originally developed for conventional angiography, this classification framework has been successfully adapted for CCTA imaging, maintaining its clinical utility in the advanced imaging era. Precise classification of PDA morphology using this system is crucial for determining the technical feasibility of transcatheter closure and selecting the most appropriate occlusion device [62]. Type A morphology with adequate ampulla is most amenable to standard Amplatzer Duct Occluder device with high success rates, while Types B (window) and C (tubular) lacking sufficient ampulla may require vascular plugs or surgical intervention if adequate anchoring cannot be achieved [38, 64]. Type E (elongated) ducts remain suitable for percutaneous closure with appropriate device selection [64].

**Table 3.** **The Krichenko classification of patent ductus arteriosus morphology based on angiographic appearance and their clinical implications [61, 62, 63]**.

PDA type	Morphological features	Clinical significance	Schematic	2D	3D
Type A (Conical)	∙ Well-defined aortic ampulla	Most suitable for standard device closure techniques	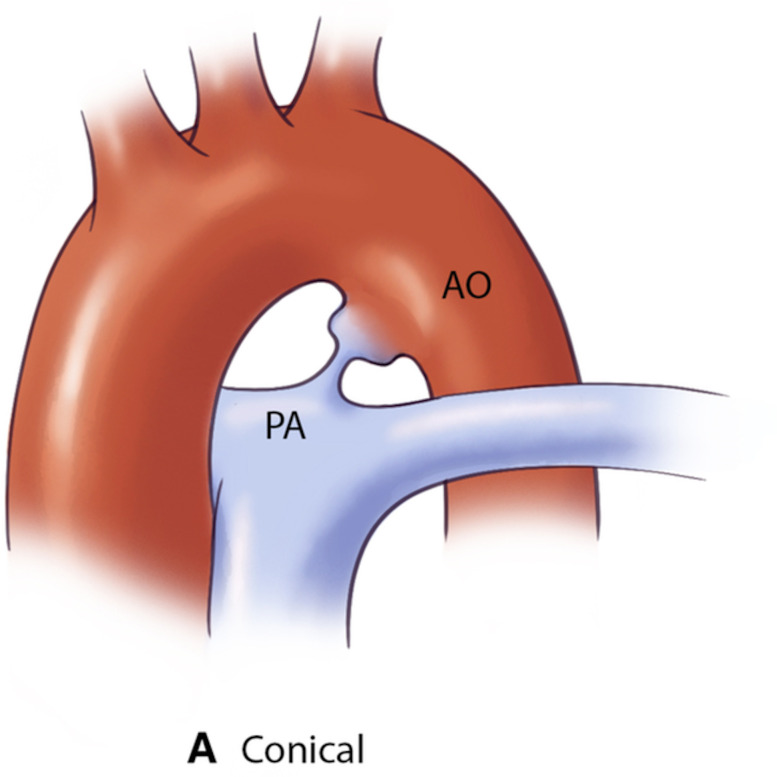	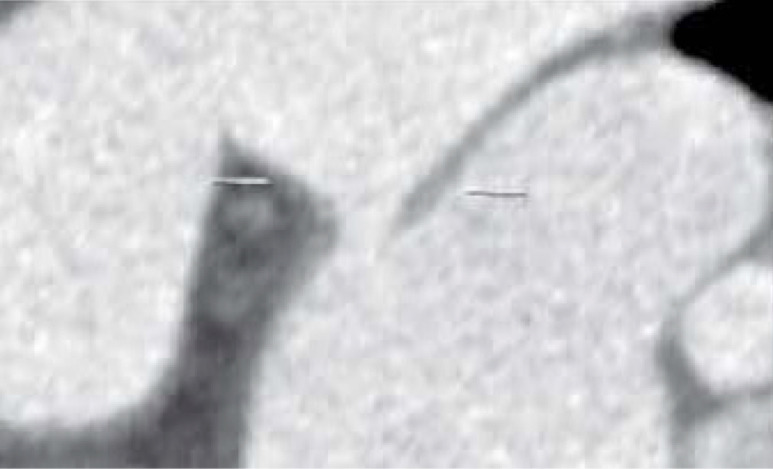	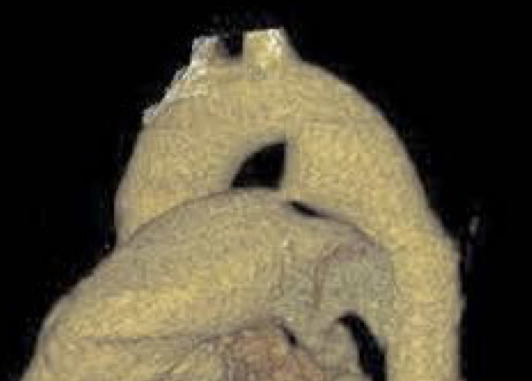
	∙ Constriction at pulmonary end				
	∙ Most common variant				
Type B (Window)	∙ Very short length with constricted aortic end	Not amenable to transcatheter closure in adults	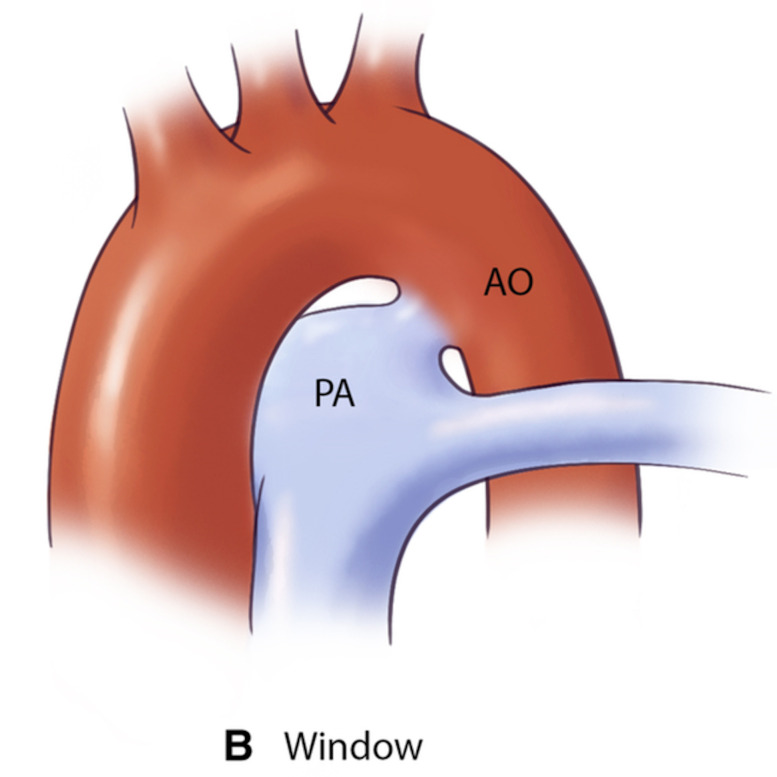	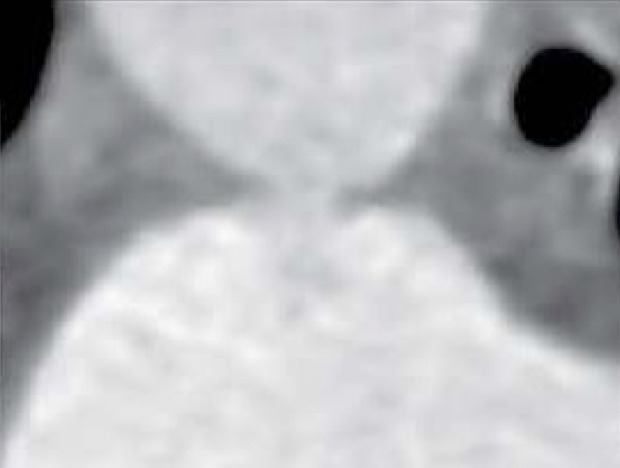	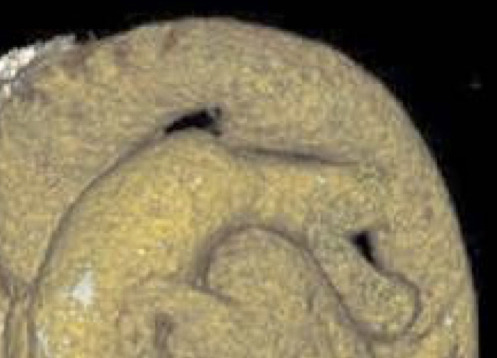
	∙ Wide pulmonary end				
Type C (Tubular)	∙ No significant constriction	May require careful device sizing to ensure stability	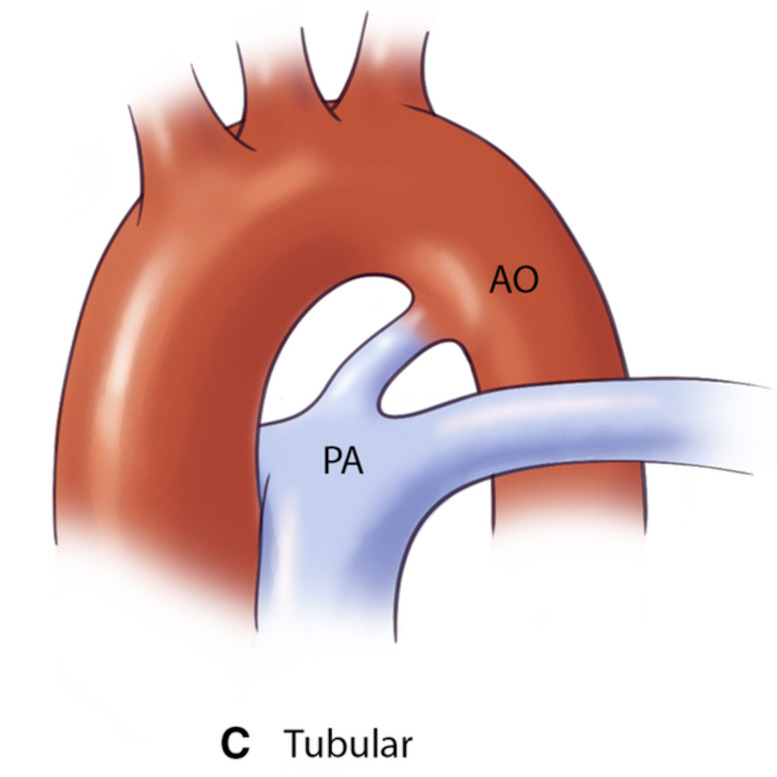	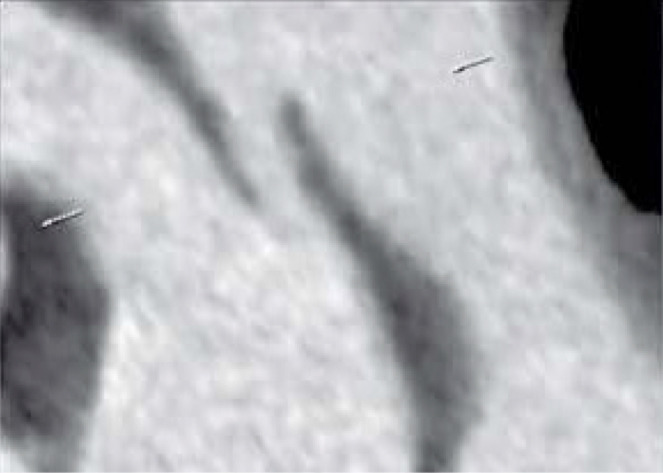	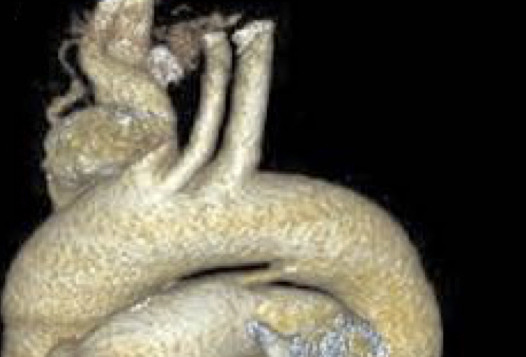
	∙ Uniform diameter throughout				
	∙ Similar width at both ends				
Type D (Complex)	∙ Multiple constrictions	Requires careful evaluation for appropriate device selection	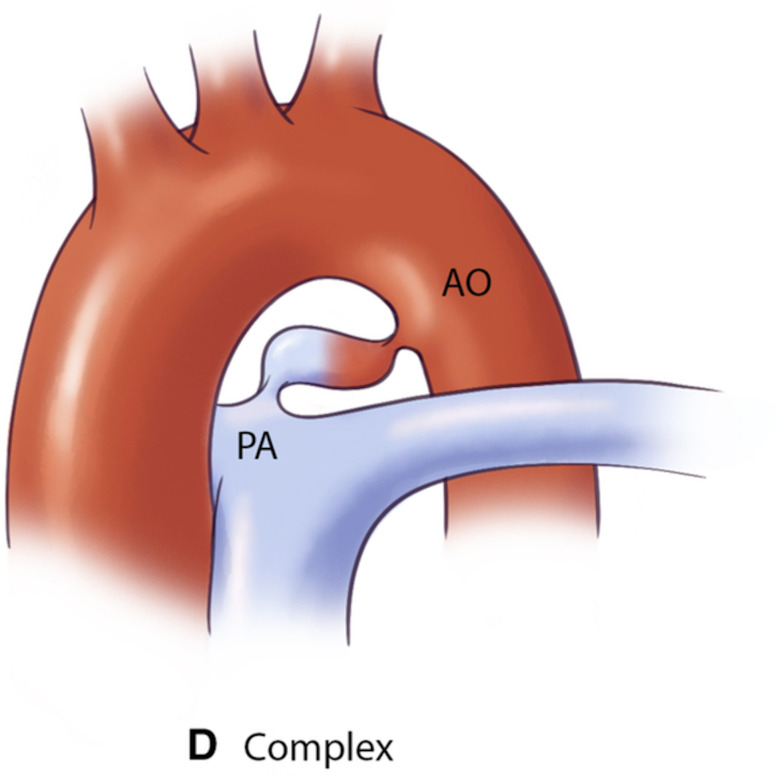	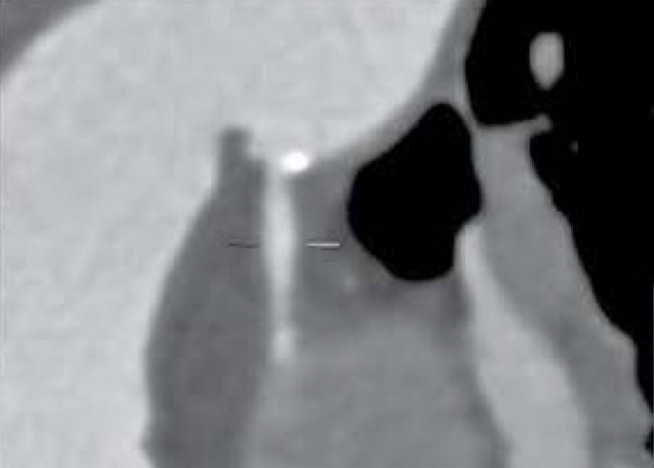	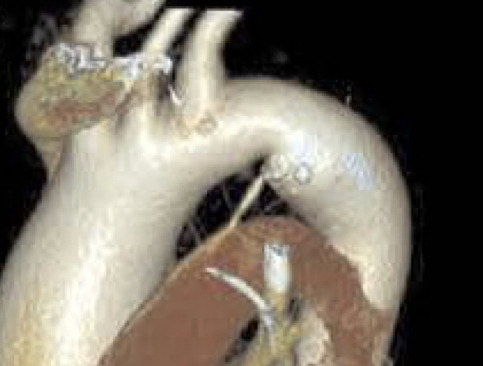
	∙ Narrow aortic and pulmonary ends				
	∙ Dilated central portion				
Type E (Elongated)	∙ Long, tortuous course	May present technical challenges for device positioning	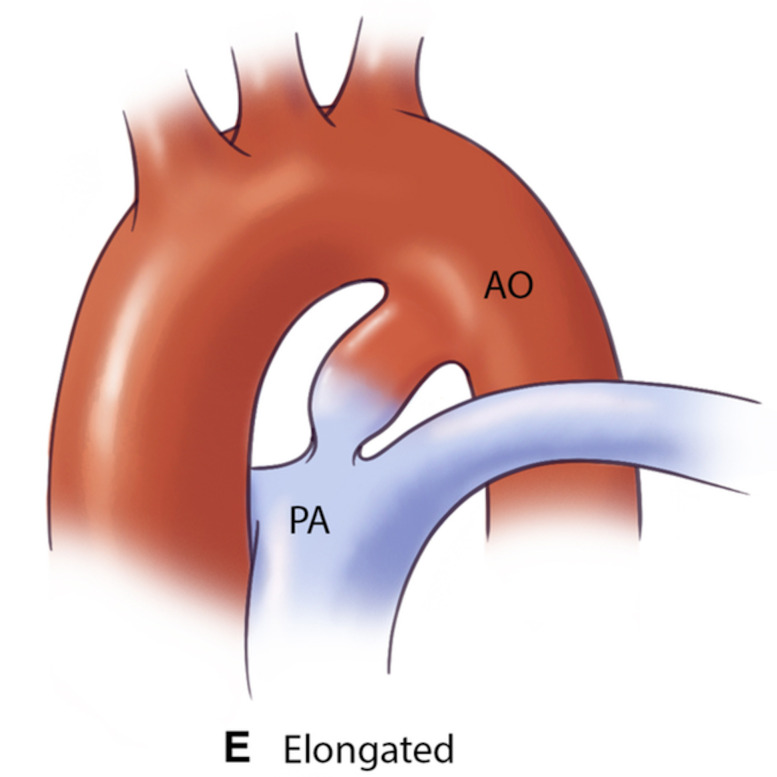	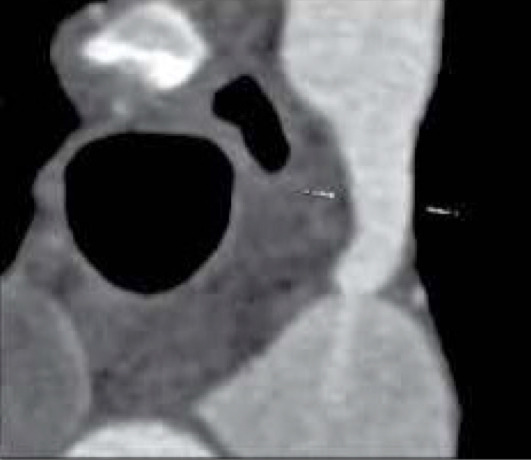	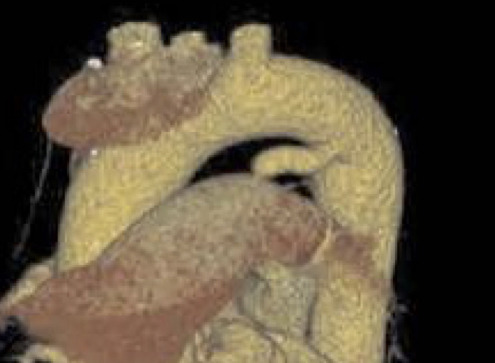
	∙ Constriction near pulmonary end				
	∙ Convoluted path between vessels				

AO, aorta.

Despite the historical role of conventional angiography in PDA evaluation, this modality’s two-dimensional nature limits the precise measurement of ductal dimensions, potentially leading to complications such as device embolization [65, 66]. ECG-gated CCTA overcomes these limitations through comprehensive 3D assessment capabilities, including determination of ductal length, measurement of minimal and maximal diameters, and morphological classification. These parameters are crucial for optimal device selection during pre-procedural planning [62, 67, 68, 69]. CCTA-derived measurements directly inform procedural decisions in adult PDA management. The minimal ductal diameter guides device selection, with larger diameters combined with shorter length increasing embolization risk and requiring carefully measured device oversizing [70]. Ductal length assessment identifies short ducts that limit landing zones, potentially contraindicating percutaneous closure [70]. Calcification, readily detected on CCTA, significantly impacts procedural planning [71]. While moderate calcification often favors transcatheter approaches given that surgery is “potentially hazardous” in adults per ACC/American Heart Association (AHA) guidelines, severely calcified or “window-like” ducts may resist device anchoring, necessitating surgical consideration [72]. Consequently, CCTA should be considered in individuals with PDA not only for primary diagnosis but also to evaluate extra-cardiac anatomy including lung parenchyma and pulmonary vasculature [73]. Beyond its diagnostic utility in the pre-procedural setting, CCTA maintains significant clinical utility in the post-intervention phase of PDA management. It enables comprehensive evaluation of several critical parameters that directly impact clinical outcomes: (1) device morphology and position relative to adjacent vascular structures; (2) potential mechanical complications including device migration, embolization, or deformation; (3) hemodynamic sequelae such as pulmonary artery or aortic stenosis; (4) thrombotic complications associated with the occlusion device; (5) infectious complications including endarteritis; and (6) residual shunting with potential hemolysis [58, 69, 74].

Standardized post-procedure imaging protocols should include thin-slice (≤1 mm) acquisition with multiphase reconstruction to capture both systolic and diastolic phases, particularly when evaluating for residual shunting or vascular stenosis. To optimally visualize PDA anatomy, specific attention to acquisition protocols is essential. The recommended imaging range extends from the aortic arch to the diaphragm, facilitating comprehensive visualization of the entire relevant vascular territory [73, 75]. To ensure detection of small PDAs, which can vary significantly in size, thin collimation techniques are strongly recommended [73, 75]. Contrast bolus timing synchronized to aortic opacification provides ideal enhancement for standard aortic arch imaging, enabling accurate delineation of ductal anatomy [75]. In complex cases with varied filling patterns (including retrograde filling of the ascending aorta, anterograde filling of the descending aorta through the PDA, or combined patterns), image acquisition timing can be strategically adjusted to either the pulmonary artery or descending aorta, selecting whichever structure demonstrates optimal clarity on the monitoring sequence. Dynamic flow assessment can be achieved through careful attention to contrast timing, with two distinct patterns observable: a “negative jet” representing unenhanced blood flow from aorta to pulmonary artery, and a “positive jet” demonstrating enhanced blood flowing from aorta to unenhanced pulmonary artery [61].

**Fig. 3.**
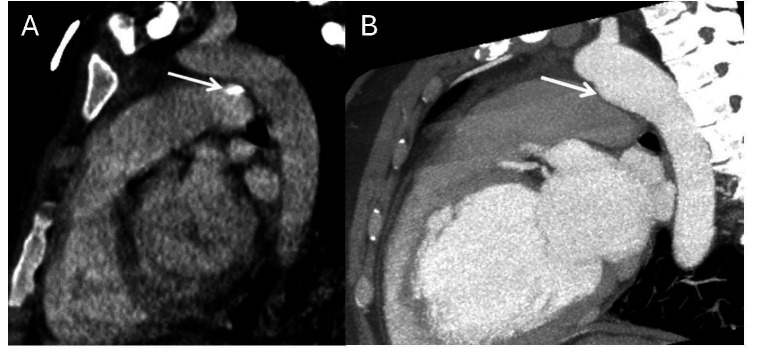
**Normal anatomic variants that may mimic PDA on imaging**. (A) Contrast-enhanced chest CT with oblique reformatting reveals calcification of the ligamentum arteriosum at its typical location between the aortic isthmus and proximal left pulmonary artery. (B) Contrast-enhanced chest CT with oblique reformatting in a different patient shows a ductus diverticulum (arrow), representing a residual aortic outpouching that occurs when the ductus arteriosus undergoes its normal closure pattern beginning from the pulmonary arterial end [77].

Several important technical and anatomical considerations must be recognized when evaluating PDAs on CCTA. Conically-shaped PDAs frequently demonstrate a characteristic linear valve-like structure at the pulmonic end, which should be recognized as a normal finding [58]. Beyond these normal variants, two common diagnostic pitfalls warrant specific attention during image interpretation: first, calcification within the ligamentum arteriosum may mimic a small PDA on contrast-enhanced studies, necessitating correlation with non-enhanced images for accurate differentiation [58]. Second, a ductus diverticulum can closely resemble a conically-shaped PDA on initial review, but can be distinguished by the absence of a connection to the left main pulmonary artery [58, 76] (Fig. 3, Ref. [77]). Recognition of these potential misinterpretations is essential for accurate diagnosis and appropriate clinical management.

## 6. Anomalous Pulmonary Venous Return

Anomalous pulmonary venous return occurs when one or more pulmonary veins (PV) fail to drain into the left atrium (LA), accounting for 1.5–5% of all congenital heart defects [78]. APVR can be either total (TAPVR) or partial (PAPVR).

### 6.1 Total Anomalous Pulmonary Venous Return

TAPVR can be divided into four subclassifications based on the location of the connections between the PV and the right-sided systemic circulation: supracardiac, cardiac, infracardiac, and mixed [79]. This section will outline the anatomy of the various subtypes of TAPVR seen on CCTA.

**Fig. 4.**
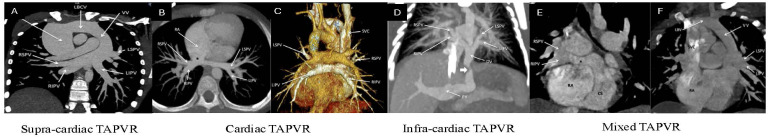
**CCTA images delineating various types of total anomalous pulmonary venous return (TAPVR)**. (A) Supracardiac TAPVR. All four PVs can clearly be seen draining into the vertical vein (VV), draining into the left brachiocephalic vein (LBCV), and ending their course into the SV. (B) Cardiac TAPVR. All four PVs are draining directly into the RA. (C) Cardiac TAPVR volume-rendered 3D image of the posterior view showcasing this anomaly. (D) Infracardiac TAPVR. All four pulmonary veins coalesce into a descending vein (DV) which travels through the esophageal hiatus and drains into the portal vein (PV). (E) Mixed TAPVR. Axial view showcasing the right superior and inferior pulmonary veins draining into the right atrium via the coronary sinus. (F) Mixed TAPVR Coronal view of the same patient showcasing the left-sided pulmonary veins draining into the right atrium via a VV and LBCV [16]. RSPV, right superior pulmonary vein; SVC, superior vena cava; RIPV, right inferior pulmonary vein; LIPV, left inferior pulmonary vein; LSPV, left superior pulmonary vein; LBV, left brachiocephalic vein.

The supracardiac type is the most common form of TAPVR, accounting for 45% of cases, and is formed when all 4 PVs drain into a confluence from which a vertical vein (VV) emerges. The VV drains into the left brachiocephalic vein (LBCV), ending its course into the superior vena cava (SVC) [79]. In this subtype, obstruction may occur at either the origin or site of drainage of the VV into the LBCV [16].​ The cardiac type is the second most common subtype of TAPVR, accounting for 15–30% of cases [79]. On CCTA, the PV can be seen draining directly into the posterior wall of the right atrium or into the coronary sinus as a conduit to the RA (Fig. 4, Ref. [16]). In the infracardiac type, the confluence of the PVs gives rise to a descending vein that traverses through the esophageal hiatus and drains into the infradiaphragmatic systemic veins. This involves connections most commonly to the portal venous system but can also involve the azygous system, hepatic vein, or inferior vena cava (IVC) [16]. This type of TAPVR is the most common to undergo pulmonary venous obstruction in up to 78% of patients, likely due to the extrinsic narrowing and resultant compression from the diaphragm [16, 79]. The mixed type of TAPVR comprises 2–10% of cases and manifests as pulmonary venous drainage into at least two locations. The most common pattern consists of the VV draining into the LBCV and drainage of the right lung (via the right pulmonary veins) into the right atrium or the coronary sinus [79].

### 6.2 Partial Anomalous Pulmonary Venous Return

**Fig. 5.**
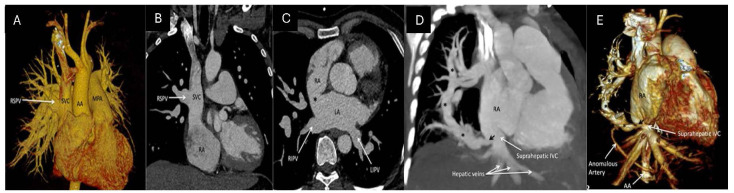
**PAPVR and scimitar syndrome on CCTA: **(A) Volume-rendered 3D image of RSPV draining into the SVC. (B) with a corresponding 2D coronal image. (C) An axial image showcases a sinus venosus ASD (*) with a normal drainage pattern in the remaining bilateral inferior pulmonary veins. (D) Scimitar syndrome highlighting the anomalous right pulmonary vein (*) draining the right lung into the suprahepatic portion of the inferior vena cava (IVC). (E) The volume-rendered 3D image showcases the anomalous pulmonary vein (*) draining into the suprahepatic IVC [16].

The prevalence of PAPVR is 0.4–0.7% and occurs when one to three PVs have anomalous drainage, with the most involved vein being the right superior pulmonary vein (RSPV) draining to the SVC or directly to the RA [78, 79, 80]. As previously stated, PAPVR can also be divided into the same types as TAPVR, with the mixed type creating a heterogeneous combination of drainage patterns, including one in which at least one vein drains into a different venous compartment [80]. PAPVR tends to result in a left-to-right shunt and becomes clinically significant when at least 50% of the pulmonary blood returns anomalously [79]. Pulmonary abnormalities associated with PAPVR include right lung hypoplasia, malformations of the right pulmonary artery (PA) and bronchial tree, and pulmonary sequestration of the right lung. *Scimitar syndrome* is a type of PAPVR that involves abnormal drainage of the right-sided pulmonary veins directly into the supradiaphragmatic or infradiaphragmatic IVC and is often associated with right lung hypoplasia [78, 79, 81] (Fig. 5, Ref. [16]). Due to its association with the IVC, CCTA (as opposed to echocardiography) is often the best modality for anatomical assessment of the right-sided PV drainage, and MRI is often subsequently used to calculate the Qp/Qs, with a ratio ≥1.5 in a symptomatic patient being an indication for surgical intervention [81].

### 6.3 Role of CCTA for APVR Assessment

CCTA has multiple apparent advantages over other modalities for the assessment of APVR. While MRI is useful for assessing anomalous pulmonary veins, it has limited spatial resolution compared to CT. White-blood imaging sequences may not adequately visualize peripheral pulmonary veins, and even with contrast or time-of-flight magnetic resonance angiography (MRA), visualization remains limited. MRA’s phase-contrast imaging can evaluate blood flow patterns, but it generally requires longer imaging times than CCTA [82, 83, 84]. In cases where extra-cardiac lung abnormalities, such as horseshoe or hypoplastic lung, coexist in patients with APVR, CCTA can reliably detect and assess these anomalies, along with accurately characterizing the anatomy of APVR [82, 83, 85]. Moreover, while echocardiography is an excellent primary screening modality to raise suspicion for APVR due to its ability to detect hemodynamic and structural abnormalities noninvasively, its evaluation for PAPVR is often nonconclusive. Echocardiography also has low diagnostic sensitivity in TAPVR with right isomerism (associated with 31% of cases of TAPVR) [78]. In contrast, the utilization of CCTA pre-procedurally allows for detailed measurements of pulmonary vein ostia, precise delineation of abnormal connections, detection of obstruction sites, and the assessment of associated cardiac and extra-cardiac anomalies (highlighted in Table 2) [75]. This information is crucial for evaluating pre-operative surgical risk, as Karamlou *et al*. [86] demonstrated increased post-surgical mortality in patients with infra-cardiac and cardiac APVR, along with those with pulmonary venous obstruction. Beyond risk stratification, CCTA aids surgical planning by visualizing abnormal drainage patterns. For example, when pulmonary veins drain away from the left atrium, as in scimitar syndrome or anomalous return to the SVC, surgeons may need to utilize pericardial rolls and baffles to minimize the risk of stenosis and obstruction [87]. Additionally, in cases where pulmonary veins drain into the right SVC above the cavoatrial junction and a sinus venosus ASD is present, the Warden procedure is advantageous. This technique reduces manipulation near the sinus node by transecting the SVC above the anomalous insertion and connecting it to the right atrial appendage. The pulmonary venous return from the lower SVC segment is then redirected through the ASD into the left atrium, followed by patch closure of the defect just above the intracardiac SVC orifice [88]. After surgical repair of APVR, CCTA is a powerful tool that can be used to monitor the patency of the anastomosis sites or identify direct or indirect signs of residual pulmonary venous obstruction [78].

For CCTA evaluation in most cases of APVR, it is generally preferred to inject contrast via an IV line placed in the antecubital vein of the upper extremity. Due to the complex anatomy of APVR, the scanning range should include structures from the thoracic inlet to the diaphragm and potentially extend to the upper abdomen if there is a suspicion for infra-diaphragmatic TAPVR [16]. Image acquisition should be timed to the pulmonary artery or left atrium to ensure optimal opacification of the pulmonary veins. In cases of obstruction or mixed APVR, longer contrast injections or delayed acquisition may be required [75]. When analyzing a CCTA study for APVR, it is imperative to consider several key factors (Table 2). Attention should be directed towards the specific anatomical connections of the anomalous veins, as well as any coexisting cardiac anomalies, such as ASD, which frequently occur in cases of APVR. Additionally, it is imperative to assess the diameter of each PV at its origin and at potential sites of stenosis, as this information is critical for informing surgical planning [16, 75, 79]. Lastly, in contrast to smaller cardiac shunts, the assessment of APVR is usually sufficient without ECG-gated scanning. Therefore, aggressive dose reduction strategies (highlighted in Section 2 above) should be used frequently to minimize radiation [75, 89].

## 7. Value of CCTA in Assessment of Rare Shunts

Cardiac CT technology not only provides 3D anatomical evaluation, but also functional and valvular assessment, which is vital for the visualization and diagnosis of relatively rare congenital heart diseases involving shunts such as coronary artery fistulas and unroofed coronary sinus.

### 7.1 CCTA and Coronary Artery Fistulas

**Fig. 6.**
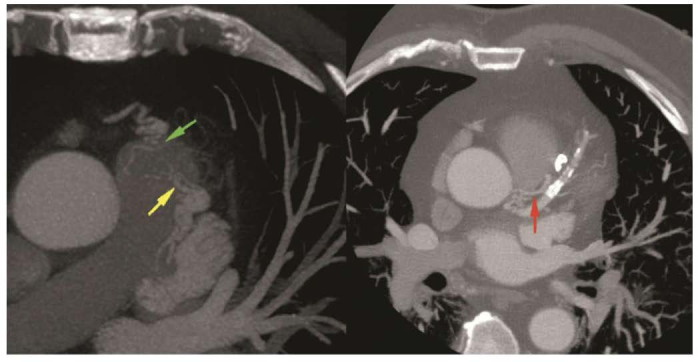
**Cardiac computed tomography angiography images revealing: CAF draining into the PA axial MIP**. A fistulous tract noted (green and yellow arrows) between LAD, conus branch and PA. The red arrow denotes a fistulous tract into the pulmonary artery from the left main coronary artery. Figure adapted and reprinted, with permission [90]. MIP, maximum intensity projection; LAD, left anterior descending artery.

**Fig. 7.**
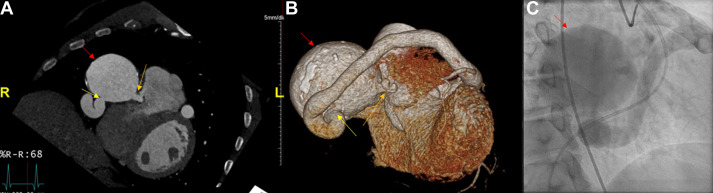
**Large coronary cameral fistula and single coronary artery demonstrated by CCTA and catheterization**. (A) CCTA of heart and coronary arteries depicting coronary artery aneurysm (red arrow) terminating as a coronary cameral fistula into the right ventricle (orange arrow) and the ectatic single coronary artery (yellow arrow). (B) Three-dimensional reconstruction and (C) left heart catheterization image revealing the same. Figure adapted and reprinted, with permission [91].

CAFs (Figs. 6,7, Ref. [90, 91]) are rare coronary anomalies that result from an abnormal termination that allows blood to bypass the myocardial capillary bed and flow directly into heart chambers or major blood vessels [92]. The imaging characteristics of CAFs can be diverse and complex, while the clinical presentations are largely influenced by factors such as the fistulas’ size, origin, course, coronary communications, and drainage location [92, 93]. Therefore, precise 3D imaging evaluation via CCTA of these factors is essential for effective diagnosis, treatment planning, and follow-up assessment.

CAFs can be classified into the following two types: (1) coronary to pulmonary artery fistulas, as shown in Figs. 6,7 [90, 91, 94], and (2) coronary artery to systemic fistulas [91]. Correct diagnosis and precise evaluation are important determinants for guiding management, as there is significant heterogeneity in the anatomy, sizes, and flow rates of CAFs. Coronary angiography, often with a right heart trans-catheterization, has been the diagnostic test of choice used to evaluate the fistula’s anatomy and calculate shunt hemodynamics [93, 95, 96]. However, the limited angles of projection of the 2D images make proper evaluation and treatment challenging due to the complex configurations, multiplicity of coronary fistulas, and their convoluted origin and drainage routes. CCTA, with its noninvasive 3D anatomical depiction, helps identify CAFs and their complex vascular relationships [97]. Practical scanning considerations and protocols can vary, but often include using a standardized ECG-triggered prospective scan protocol, aiming for arterial phase contrast enhancement. A test bolus is recommended over the bolus-tracking method as it considers an individual patient’s physiological parameters, especially since the fistula can act as a large reservoir or a high-flow shunt [97, 98]. Due to the dilated nature of CAFs, sublingual nitroglycerin is contraindicated [97].

Furthermore, dual-energy CT applications, such as iodine mapping, increase contrast conspicuity of subtle or small fistulas, offer material decomposition to assess morphology, and reduce blooming artifacts to enhance CAF differentiation [98]. Depending on the termination’s location and the surgical approach, various catheters, such as pre-shaped coronary guiding catheters and deflectable sheaths, can assist in wire crossing. For larger CAFs, it is necessary to use vascular occluders with catheters that are 5-F or larger. When the fistula starts from the distal third of the coronary vessel, a transvenous approach is recommended because it reduces the risk of inadvertently damaging the parent vessel. The transarterial approach is recommended for fistulas originating from the proximal coronary, since there is a shorter distance to traverse through the parent vessel [93].

### 7.2 CCTA for Unroofed Coronary Sinus

**Fig. 8.**
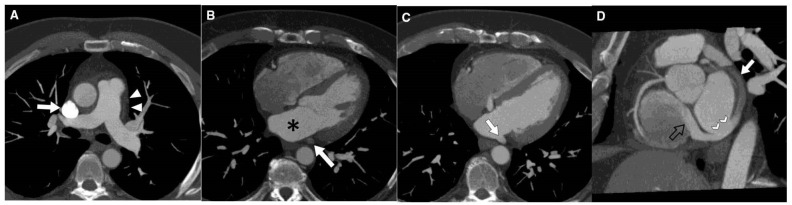
**Contrast-enhanced cardiac CT images revealing unroofed coronary sinus (CS)**. (A) Axial MIP image at PA level demonstrates a right SVC (arrow) with absence of left SVC (arrowheads). (B) Axial MIP image at midventricular level shows CS (arrow) unopacified and separated by fat plane from LA (*). (C) Axial MIP image 2 cm inferior to (B) shows CS unroofed (arrow) and with same opacification as LA. (D) Multiplanar reformatted image in valve plane shows CS partially unopacified (arrow), site of unroofing (arrowheads), and jet of dense contrast entering the right atrium through the coronary sinus valve due to the left-to-right shunt (black open arrow). Figure adapted and reprinted, with permission [100].

Unroofed coronary sinus, the rarest type of ASD, occurs when there is a partial (either focal or fenestrated) or complete absence of the atrial wall between the coronary sinus (CS) and left atrium [99] (Fig. 8, Ref. [100]). It is a rare cardiac anomaly accounting for <1% of lesions [101]. The clinical significance of a UCS depends on the drainage site and the associated cardiac abnormalities. Furthermore, if a right-to-left shunt occurs, it increases the risk of cerebral embolism and brain abscesses; thus, it is important to diagnose the disease early and accurately [13]. TTE is the most common method for diagnosing cardiac malformation in UCS. However, it is difficult to ascertain the CS defect located posterior to the heart by TTE due to its limited acoustic windows and resolution [13]. Furthermore, diagnosing a complete UCS is difficult due to its direct connection to the left atrium, which obscures its characteristic tubular structure. CCTA imaging can accurately and precisely depict anomalous vessel course, diameter, drainage sites, and termination, which is essential for effective treatment [102]. Preoperative observation of the UCS defect morphology via CCTA is vital for the decision regarding patch repair of the gap between CS and LA or ligation of the coronary sinus ostium, provided UCS is not combined with persistent left superior vena cava (PLSVC) [13].

There are four morphological types of UCS defects, classified according to the Kirklin and Barratt Boyes’s method: Type I (complete absence of parietal wall of CS with PLSVC), Type II (complete absence of parietal wall of CS without PLSVC), Type III (perforation of the middle segment of the parietal wall of CS), Type IV (perforation of the parietal wall of the end segment of CS) [13]. To visualize the UCS, the optimal CCTA scan range is from 10–15 mm below the tracheal bifurcation to the diaphragmatic surface of the heart [13]. The cardiac short-axis view in the plane of the atrioventricular groove is best for visualization of a UCS on CCTA for surgical repair, as well as differentiating between UCS subtypes [103]. Multiplanar reformation (MPR) images of the UCS can be reconstructed using this short-axis view, which shows the entire course of the UCS. The interactive evaluation of MPR images helps better understand the vascularity of the UCS and possible associated malformations in the adjacent tissue [13]. In some patients, UCS can be accompanied by a PLSVC, which results when the left superior cardinal vein caudal to the brachiocephalic vein fails to regress during development, causing a dilated coronary sinus [104]. This combination is known as Raghib syndrome [105]. Clinically, PLSVC is characterized by prevalent angiographic findings such as coronary vessel contortion, which often causes lead manipulation and placement issues in patients [14]. CCTA has emerged as a strong imaging technology with thin slices and multiplanar reformations that can help provide a detailed assessment for the evaluation of PLSVC. The optimal contrast opacification of PLSVC is mostly seen in the delayed venous phase images of CCTA, as identification is usually independent of the administered contrast injection route [105]. Furthermore, if PLSVC is combined with a UCS, the central venous pressure needs to be measured to determine if there is an obstruction in the RSVC and/or LSVC to help determine the surgical approach [13].

## 8. CCTA-Based 3D Printed Models in Pre-Procedural Evaluation of Congenital Shunt Lesion

Contemporary implementation of 3D printing technology has substantially augmented the diagnostic and interventional utility of CCTA in the evaluation of cardiac shunts. Patient-specific anatomical models derived from high-resolution CCTA datasets enable comprehensive pre-procedural assessment of defect morphology, dimensions, and spatial relationships to adjacent cardiac structures [106, 107, 108, 109]. The workflow begins with high-resolution imaging (≤1.25 mm slices), followed by segmentation and model refinement to isolate septal anatomy and relevant chambers. The choice of printing modality is tailored to the procedural goal, whether evaluating fit, flexibility, or device navigation [110, 111]. In anatomically complex ASDs and VSDs such as larger diameter defects or multiple defects, particularly those with atypical morphology or proximity to vital structures, 3D printing has emerged as a powerful tool for procedural planning. It enables patient-specific simulation of closure techniques—including patch sizing and transcatheter device deployment—within a reproducible, anatomy-matched model [112, 113]. In patients with PDA, 3D printed models can be particularly useful in complex cases with tortuous or calcified ductal anatomy [114]. Comparative studies have demonstrated excellent correlation between printed models and source imaging, supporting their reliability in clinical decision-making [115]. This technological integration represents a significant advancement in procedural planning, structural orientation, and pre-interventional simulation for the management of simple cardiac shunts in the adult population.

Despite its early promise, 3D printing brings with itself certain barriers that limit standardized inculcation in routine surgical planning. High cost of printers and specialized software remains to be a limiting factor, especially in resource-limited settings. When considering systems-level utility, workflow integration is another challenge, as producing a clinically usable model demands time-intensive and time-sensitive segmentation, post-processing, and multi-disciplinary coordination among physicians, engineers, and procedural teams [116]. These are steps that may not fit urgent surgical timelines. Inelastic printed models, while visually accurate, have still not been developed enough to fully mimic dynamic biological motion, such as predicting interventricular septal compliance during transcatheter VSD closure, which can reduce the precision of device simulation [117]. While the promise of 3D printing is certainly very exciting, the key to unlocking its potential lies in addressing these limitations, which will require faster and automated segmentation alongside wider access to capable printing facilities. As with any implantable system, the development of deformable materials that consistently represent the chemistry of living tissue in both composition and behavior is of paramount importance [118].

## 9. Guideline Recommendations for the Use of CCTA in the Evaluation of Congenital Cardiac Shunts

Societal guidelines for imaging adult CHD recommend advanced imaging techniques like CCTA or MRI in specific scenarios. CCTA is the preferred method for evaluating the pulmonary arteries, aorta, collateral vessels, and arteriovenous malformations. For patients with septal defects (ASD, VSD, atrioventricular septal defect (AVSD)) and associated anomalies, either CCTA or cardiac magnetic resonance (CMR) is indicated. CMR is particularly advantageous for assessing ventricular volumes and shunt flow due to its higher temporal resolution and lack of radiation exposure, while CCTA excels in evaluating pulmonary venous connections [38, 73, 119]. In cases of inferior sinus venosus defects, CCTA or CMR surpasses TEE for evaluation. For symptomatic patients post-Amplatzer device occlusion, CT imaging assesses atrial and venous anatomy and the patch closure area, which tends to calcify with aging. Cross-sectional imaging with CMR or CCTA effectively delineates pulmonary venous connections, especially those difficult to visualize by echocardiography, such as the innominate and vertical veins. Guidelines have recommended specific requirements for the performance of CCTA in adult CHD (See **Supplementary Table 1**).

## 10. Limitations of CCTA in the Evaluation of Shunts and Future Directions

While CTA is an exceptional tool for assessing cardiac shunts, it is not without its limitations. For example, 30–50% of patients with CHDs have concomitant renal dysfunction, which may preclude the use of iodine-based contrast in many of these patients [120]. Moreover, arrhythmias such as atrial fibrillation can result in inadequate ECG-gated studies in patients with suboptimal rate control. Additionally, scanning with thin slices to detect smaller shunts may result in long scan times with a resultant increase in radiation exposure over time, especially when serial assessments are needed [9, 121, 122]. Table 4 (Ref. [9, 39, 46, 58, 91, 121, 122, 123, 124, 125, 126, 127, 128, 129, 130, 131, 132, 133, 134, 135, 136, 137, 138, 139, 140, 141, 142, 143]) showcases additional limitations for each shunt.

**Table 4.** **Strengths and limitations of cardiac CT angiography, cardiac MRI and echocardiography for the assessment of simple intracardiac shunts in adults**.

	CCTA		Echocardiography		Cardiac MRI	
Shunt type	Strengths	Limitations	Strengths	Limitations	Strengths	Limitations
General [91, 123, 124, 125]	- High spatial and temporal resolution	- Radiation	- No radiation	- Limited assessment of extra-cardiac abnormalities associated with some types of CHD	- No radiation	- Limited availability
	- Excellent 3D reconstruction capabilities	- Iodine allergy + renal dysfunction preclude use	- Wide availability	- Quality is operator dependent	- High temporal resolution	- Longer scan times
		- Requires rate control for ECG-gated studies	- Real-time hemodynamic assessment		- Excellent hemodynamic quantification of shunts	- May require sedation for claustrophobia
		- Limited assessment of hemodynamics				- Implants or devices may preclude use
		- Longer scan times for small shunts				
PFO [9, 121, 122, 143]	- Guides preprocedural planning by providing an accurate assessment of measurements and dimensions	- Lower sensitivity and specificity for detection compared to TEE	- Excellent detection with contrast and Valsalva	- Unable to directly measure degree of shunt fraction	- Able to quantify shunt fraction with phase contrast	- Inferior to TEE in detection of contrast-enhanced right-to-left shunting and identification of atrial septal aneurysm
		- Unable to perform Valsalva to assess for right-to-left shunt	- Real-time procedural guidance for device implantation			
		- Interatrial free flap valve can be mistaken for PFO				
ASD [124, 126, 127, 128, 129, 130, 131, 132]	- Evaluates defect location, measures dimensions, and assesses surrounding rims	- Less sensitive compared to echocardiography	- First line modality for size assessment and assessing adequate rims for device closure	- May miss SVASD due to posterior location (TTE), requiring invasive TEE for diagnosis	- Correlates well with cardiac catheterization for shunt quantification	- Cannot reliably exclude small ASD
	- Advantage in the assessment of large secundum defects with deficient inferior rims		- Real-time procedural guidance for device implantation		- Reliable in ASD evaluation if echocardiographic assessment is suboptimal	
PDA [58]	- Accurate assessment of shunt patency and direction with positive/negative jet visualization	- High (13.7%) false-negative rates for silent PDA on routine 3 mm chest CT	- Primary modality of choice for diagnosis	- May sometimes miss large PDAs in the presence of pulmonary hypertension	- Able to quantify shunt fraction with phase contrast	- General limitations apply
					- Adequate hemodynamic evaluation of small PDAs	
					- Non-traditional off-axis or orthogonal places facilitate precise anatomic delineation	
VSD [39, 46, 131, 133, 134, 135, 136]	-Volume rendering simulates virtual patch closure and device placements, aiding in pre-procedural planning	- Small or irregularly bordered VSDs may be missed	- Primary modality of choice for diagnosis	- May miss small VSDs if there are poor acoustic windows	- May be useful in the diagnosis of apical VSDs	- Does not add much information to that obtained from echocardiography unless the VSD is associated with complex anomalies
		- Minor membranous VSDs may be missed	- Accurate assessment of shunt volume and fraction	- Apical defects may be difficult to visualize		
Other shunts [137, 138, 139]	- Identifies complex vascular relationships in coronary artery fistulas	- Difficulty assessing small or distal parts of certain types of coronary artery fistulas	- May be used for initial evaluation to visualize fistula connections to the heart chambers	- Limited visualization of coronary arteries	- Cine MRI can assess for flow turbulence at the fistula entry site	- General limitations apply
					- Black blood imaging allows for better visualization of the coronary lumen and wall	
APVR [131, 140, 141, 142]	- Does not require ECG-gating, thereby minimizing radiation while also providing excellent spatial resolution	- Suboptimal images may preclude use of retrospective gating for shunt fraction assessment	- High diagnostic sensitivity in isolated TAPVR (81%)	- Low diagnostic sensitivity in heterotaxy (27%) and mixed variates (20%) of TAPVR	- Accurate measurement of flow via phase contrast imaging, correlating well with invasive catheterization	- Longer than normal scan times if suspicion for infradiaphragmatic TAPVR
		- May miss extracardiac shunts if a dedicated CCTA is only performed			- Indicated in patients with isolated right ventricular dilation to exclude PAPVR	

Abbreviations: PDA, patent ductus arteriousus; MRI, magnetic resonance imaging.

Despite these limitations, recent advancements in CT technology (i.e., advanced reconstruction and artificial intelligence) have led to the increased availability of scanners with high temporal and spatial resolution. As a result, these advancements have not only reduced artifacts but also minimized radiation exposure through effective dose reduction strategies. A recent study by Dirrichs *et al*. [144] assessed the image quality and radiation exposures of first-generation photon counting CT (PCCT) scanners compared to third-generation energy-integrating dual-source CT (DSCT) in a cohort of 113 consecutive children. The findings revealed that PCCT offered a higher signal-to-noise ratio (SNR), a superior contrast-to-noise ratio (CNR), and overall better image quality, including improved sharpness, contrast, and delineation of vascular structures. Notably, there was no significant difference in the mean effective radiation dose between the two modalities. These technological advancements provide practical benefits for 3D printing, facilitating the creation of photorealistic 3D images that are invaluable for surgical planning [144].

## 11. Conclusion

CCTA offers an efficient and comprehensive analysis of various cardiac shunts. Its extensive utility enables timely pre-procedural planning, provides intraprocedural guidance, and ensures post-procedural monitoring to assess the adequacy of repairs and identify potential complications. As technology advances, CCTA is poised to enhance its value and may even surpass some of its current limitations in shunt evaluation.

## Abbreviations

CCTA, cardiovascular computed tomography angiography; IVC, inferior vena cava; CHD, congenital heart disease; SVC, superior vena cava; CT, computed tomography; LAD, left anterior descending artery; MRI, magnetic resonance imaging; RCA, right coronary artery; PCD, photon-counting detector; IAS, interatrial septum; PFO, patent foramen ovale; AV, atrioventricular; ASD, atrial septal defects; LA, left atrium; VSD, ventricular septal defects; RA, right atrium; PDA, patent ductus arteriosus; LV, left ventricle; APVR, anomalous pulmonary venous return; PA, pulmonary artery; TAPVR, total anomalous pulmonary venous return; PV, pulmonary vein; PAPVR, partial anomalous pulmonary venous return; VV, vertical vein; CAFs, coronary artery fistulas; LBCV, left brachiocephalic vein; UCS, unroofed coronary sinus; RSPV, right superior pulmonary vein; PLSVC, persistent left superior vena cava; MIP, maximum intensity projection; ECG or EKG, electrocardiogram; CS, coronary sinus; 2D, 2-dimensional; RVH, right ventricular hypertrophy; 3D, 3-dimensional; IV, intravenous; TTE, transthoracic echocardiography; DHPI, double-head power injector; TEE, transesophageal echocardiography; ROI, region of interest; Qp/Qs, pulmonary-to-systemic blood flow ratio; HU, Hounsfield units; MPR, multiplanar reformations; ALARA, as low as reasonably achievable; SV, sinus venosus.

## Supplementary Material

Supplementary material associated with this article can be found, in the online version, at https://doi.org/10.31083/RCM43059.
