# Supplementary material for: Cardiovascular Computed Tomography Angiographic Assessment of Simple Cardiac Shunts in Adults
Source: Rev Cardiovasc Med. 2025 Nov 27;26(11):43059. doi: 10.31083/RCM43059 (PMC12681020; doi:10.31083/RCM43059)
Supplement: Supplementary file 1 [file 2153-8174-26-11-43059-s1.zip › Supplementary Table 1.docx]

**Supplementary Table 1: Requirement for performance of CCTA in Adult Congenital Heart Disease**

| **2020 ESC Guidelines for ACHD^115^** | **2015 Expert Consensus Document of the SCCT^68^** |
| --- | --- |
| - CCTA with modern single or dual source scanners can be performed with dose-saving protocols. - Interdisciplinary expert collaboration is important: imaging CHD experts need to respond to feedback from CHD surgeons, interventionalists, and electrophysiologists to optimize imaging contribution to care, as well as work with each other to enhance appropriate use of multimodality imaging. - Advanced imaging is usually best reserved for when patients are seen in the specialist center rather than be repeated - Echocardiography, CMR, and CCT require staff with expertise in CHD as well as in imaging, which has training and resource implications. [Within the ESC, this is recognized by a certification examination of the European Association of Cardiovascular Imaging (EACVI), separate from the standard TTE, TOE, or CMR examination, and specific to CHD] | - Alternate cardiac imaging modalities should be available so that the test with the least risk can be performed for a specific clinical indication. - Close collaboration & communication is present among surgeons, clinical cardiologists and imagers - All patient clinical information is accessible to allow understanding of the clinical indication and potential management options for the patient - Scan protocols can be designed and adjusted to extract maximum clinical information at minimum procedural risk - Technologists are experienced in cardiac CT and comfortable with varied cardiac scan modes - Easy access to pacemaker programming to allow rate and mode adjustment - Nursing support to facilitate administration of medication for heart rate control, when necessary, in patients with and without permanent pacemakers, and to provide appropriate monitoring for any side effects - Access to all forms of prior imaging (echocardiography, angiography, nuclear, CMR) so that a targeted evaluation may be performed for an individual patient - Post processing workstations capable of handling large multiphase data sets for advanced reconstructions - High-speed network to transfer large volume data sets from scanner to workstation - Immediate availability of advanced resuscitation equipment and resuscitation team appropriate for the size and age of the patient |
